# When genes move, genomes collide

**DOI:** 10.1371/journal.pgen.1007286

**Published:** 2018-04-12

**Authors:** Yaniv Brandvain, Daniel R. Matute

**Affiliations:** 1 Department of Plant and Microbial Biology, University of Minnesota, St. Paul, Minnesota, United States of America; 2 Biology Department, University of North Carolina, Chapel Hill, North Carolina, United States of America; Fred Hutchinson Cancer Research Center, UNITED STATES

The ultimate test of whether two diverged populations are, in fact, good species is whether they will maintain their distinctness in sympatry [1, 2]. This requires the action of one or more reproductive isolating mechanisms. Identifying traits and alleles that underlie reproductive isolation has therefore been a major focus of speciation genetics research [3, 4]. In addition to informing the basis of reproductive isolation, alleles underlying interspecific incompatibilities can provide information concerning how functional evolutionary divergence unfolds.

The genus *Mimulus* has long served as a model for the study of reproductive isolation [5, 6]. Much of this work has focused on *Mimulus guttatus* and *M*. *nasutus*. These recently diverged species have broadly overlapping geographic ranges, and—when sympatric—*M*. *nasutus*’ ancestry frequently introgresses into *M*. *guttatus* [7–9]. Because hybridization and introgression in this species pair are common, the genetic mechanisms preventing their fusion is of great interest. In this issue, Zuellig and Sweigart [10] map the genetic basis of an inviability phenotype that only manifests in F2 *M*. *nasutus* × *M*. *guttatus* hybrids.

Zuellig and Sweigart [10] identify the precise alleles of hybrid seed inviability in a pair of recently related and naturally hybridizing species of *Mimulus* (Fig 1A). After two bulked segregant analyses to identify the incompatibilities, and some impressive snooping in the unassembled portion of the *M*. *guttatus* reference genome, Zuellig and Sweigart identify 2 genes involved in the defect: *hl13* (Migut.M02023) and *hl14* (Migut.O00467). These genes are duplicates of plastid transcriptionally active chromosome 14 (*pTAC14*)―a gene known to be essential for proper chloroplast development in *Arabidopsis thaliana* [11]. A functional copy of *pTAC14* is found on chromosome 14 (*hl14*) of both the *M*. *guttatus* reference genome and the *M*. *guttatus* studied samples, but the gene is missing from *M*. *nasutus*’ chromosome 14. In contrast, both species have a syntenic copy of *pTAC14* (*hl13*) on chromosome 13, but the *M*. *guttatus* allele includes a frameshift mutation and is not expressed. As a result, some F2 hybrids and advanced backcrossed seedlings will be homozygous for the nonfunctional *M*. *guttatus*’ copy of *pTAC14* at *hl13* and *M*. *nasutus*’ null allele on chromosome 14; these individuals will lack a functional *pTAC14* gene, leaving them unable to photosynthesize and therefore inviable (Fig 1B).

**Fig 1:**
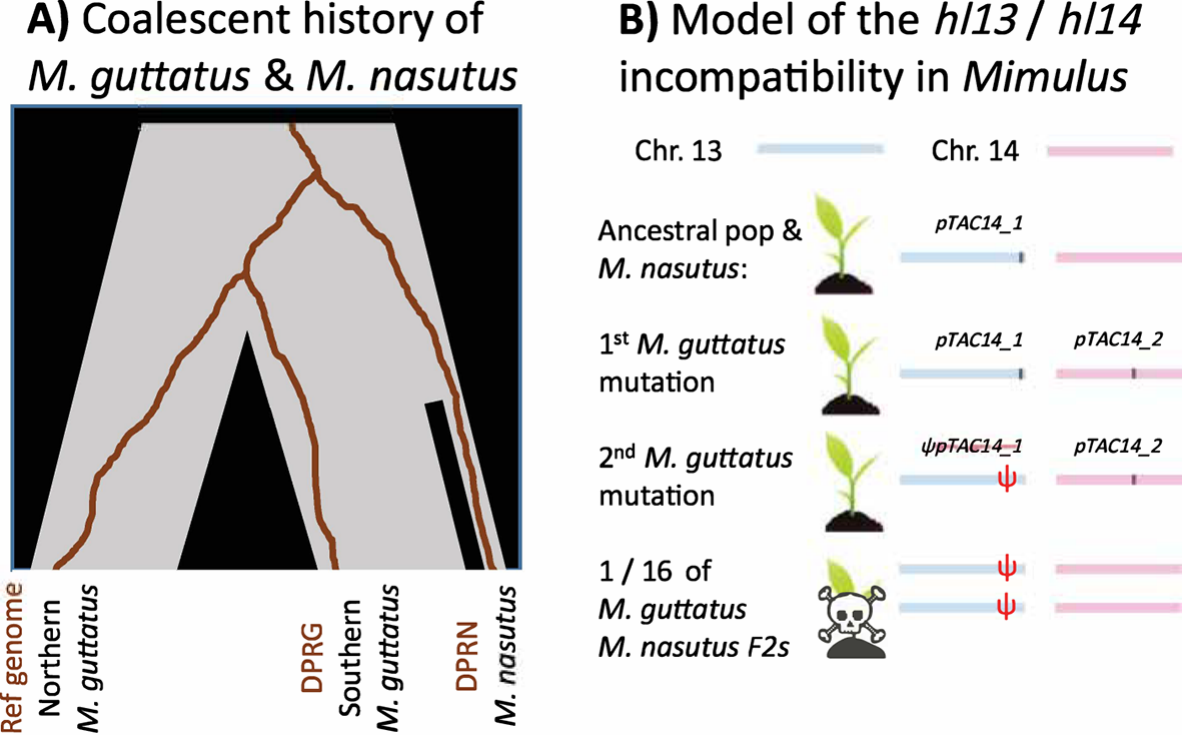
The history of the *M*. *guttatus*–*M*. *nasutus* species pair, and the evolution of the *hl13*/*hl14* incompatibility. (A) Southern *M*. *guttatus* populations (including the sample DPRG studied by Zuellig and Sweigart) are more closely related to *M*. *nasutus* (sample DPRN) than to northern *M*. *guttatus* (including the *M*. *guttatus* reference genome). Nonetheless, DPRG is more similar to the reference strain at *hl13* and *hl14*—the loci underlying the hybrid incompatibility identified by Zuellig and Sweigart—than *M*. *nasutus* (shown by the brown coalescent genealogies). (B) Zuellig and Sweigart found that (1) *pTAC14* was ancestrally located in chromosome 13 in *Mimulus*, (2) a copy moved to chromosome 14 in *M*. *guttatus*, and (3) the initial copy then lost function in *M*. *guttatus*. While all F1s are viable, one-sixteenth of F2s inherit a chromosome 14 without *pTAC14* and a chromosome 13 with a nonfunctional *pTAC14*. Because *pTAC14* is a critical photosynthetic gene, these seedlings are inviable.

The idea that divergent resolution of gene duplicates could result in the inviability of hybrids inheriting null alleles has been long hypothesized by theory [12–16]. The results are an intuitive consequence of gene movement and meiotic segregation. Moyle et al. [12] first formalized this model of stepwise gene movement leading to hybrid incompatibility―first a gene is duplicated, then it loses function in its original location, and, effectively, the only remaining copy is in a different chromosome. The nature of Mendelian segregation ensures hybrid defects that affect only a proportion of hybrids (in this case no F1 is affected, while one-sixteenth of the F2s are).

Previous studies also have lent support for this model. In the *Drosophila simulans*–*D*. *melanogaster* species pair, the transposition of the gene *JYalpha* from the fourth to the third chromosome in *D*. *simulans* causes sterility in the hybrid males [17]. A similar result has been found between accessions of the selfing plant *A*. *thaliana* from Columbia (Col) and Cape Verde Island (Cvi)—the histidinol-phosphate aminotransferasegene exists on chromosome 1 but not chromosome 5 in Cvi, and on chromosome 5 but not chromosome 1 in Col, and F2s lacking histidinol-phosphate aminotransferasegene are inviable [18]. Unlike *M*. *guttatus* and *M*. *nasutus*, none of these species pairs hybridize in nature.

The results from Zuellig and Sweigart have three important implications, each offering new research directions. First, this study will allow researchers to ask how incompatibility due to gene movement contributes to genome variation and differentiation in natural populations. For example, future studies and/or reexamination of genomic patterns of introgression between these species [9] could examine whether *M*. *nasutus*’ ancestry is elevated around *hl14* and depleted around *hl13* in sympatric *M*. *guttatus*, as would be expected if incompatible alleles are selected against upon introgression [19–21]. Additional directions could address how *hl13/hl14* acts in concert with the other reproductive isolating mechanisms and mapped incompatibilities (e.g., [22]) to prevent the fusion of these species in sympatry.

Second, both the process of duplication of *pTAC14* and nonfunctionalization are plausibly neutral, suggesting that reproductive isolation may have arisen by a neutral process. In fact, Zuellig and Sweigart find no strong evidence that natural selection is responsible for either *pTAC14*‘s duplication or degeneration, and as such the authors suggest that this incompatibility has a neutral origin. While this hypothesis is plausible, it clearly needs more scrutiny. Further study of the effects of the alternative functional copies, and additional population genomic studies of the history of selection on *hl13* and *hl14*, could inform the evolutionary question of how and why genes relocate.

Finally, the recency of the split between these species and the extensive natural variation in *M*. *guttatus* mean that the *hl13/hl14* incompatibility provides an excellent opportunity to identify the factors shaping the alternative resolution of alternative paralogs. This question is particularly interesting because of the evolutionary history of the species’ split; the *M*. *guttatus* population studied by Zuellig and Sweigart is more closely related to *M*. *nasutus* than it is to the *M*. *guttatus* reference genome with which it shares *hl13* and *hl14* alleles [8]. This observation―that the gene trees underlying hybrid incompatibilities do not match the population tree—may seem counterintuitive, but is potentially consistent with recent results from theoretical population genetics [23].

Overall, Zuellig and Sweigart’s results provide empirical evidence that the evolution of gene duplicates might be involved in reproductive isolation in young species that hybridize in nature. Systematic efforts like Zuellig and Sweigart’s will reveal to what extent gene movement is a prevalent force in the formation of species. More generally, their study provides a map route to understand what is the role of hybrid incompatibilities in keeping naturally co-occurring―and potentially hybridizing―species apart.

## References

[1] Coyne JA, Orr HA (2004) Speciation. Sunderland, MA: Sinauer Associates, Inc.

[2] Harrison RG (2012) The language of speciation. *Evolution* 66(12):3643–3657. doi: 10.1111/j.1558-5646.2012.01785.x 23206125 10.1111/j.1558-5646.2012.01785.x

[3] Maheshwari S, Barbash DA (2011) The genetics of hybrid incompatibilities. *Annu Rev Genet* 45(1):331–355. 21910629 10.1146/annurev-genet-110410-132514

[4] Nosil P, Schluter D (2011) The genes underlying the process of speciation. *Trends Ecol Evol* 26(4):160–167. doi: 10.1016/j.tree.2011.01.001 21310503 10.1016/j.tree.2011.01.001

[5] Vickery RK (1964) Barriers to gene exchange between members of the *Mimulus guttatus* complex. *Source Evol* 18(1):52–69.

[6] Wu CA, Lowry DB, Cooley AM, Wright KM, Lee YW, Willis JH (2008) *Mimulus* is an emerging model system for the integration of ecological and genomic studies. *Heredity (Edinb)* 100(2):220–230. 17551519 10.1038/sj.hdy.6801018

[7] Sweigart AL, Willis JH (2003) Patterns of nucleotide diversity in two species of *Mimulus* are affected by mating system and asymmetric introgression. *Evolution (N Y)* 57(11):2490–2506. 10.1111/j.0014-3820.2003.tb01494.x 14686526

[8] Brandvain Y, Kenney AM, Flagel L, Coop G, Sweigart AL (2014) Speciation and Introgression between *Mimulus nasutus* and *Mimulus guttatus*. *PLoS Genet* 10(6):e1004410 doi: 10.1371/journal.pgen.1004410 24967630 10.1371/journal.pgen.1004410 PMC4072524

[9] Kenney AM, Sweigart AL (2016) Reproductive isolation and introgression between sympatric *Mimulus* species. *Mol Ecol* 25(11):2499–2517. doi: 10.1111/mec.13630 27038381 10.1111/mec.13630

[10] Zuellig M, Sweigart AL (2018) Gene duplicates cause hybrid lethality between sympatric species of *Mimulus*. *PLoS Genet* 14(4): e1007130 https://doi.org/10.1371/journal.pgen.1007130 10.1371/journal.pgen.1007130 PMC5896889 29649209

[11] Gao Z-P, Yu QB, Zhao TT, Ma Q, Chen GX, Yang ZN (2011) A Functional Component of the Transcriptionally Active *Chromosome Complex*, *Arabidopsis pTAC14*, *Interacts with* pTAC12/HEMERA and Regulates Plastid Gene Expression. *Plant Physiol* 157(4):1733–1745. doi: 10.1104/pp.111.184762 22010110 10.1104/pp.111.184762 PMC3327189

[12] Moyle LC, Muir CD, Han M V., Hahn MW (2010) The contribution of gene movement to the “two rules of speciation.” *Evolution* 64(6):1541–1557. doi: 10.1111/j.1558-5646.2010.00990.x 20298429 10.1111/j.1558-5646.2010.00990.x

[13] Werth CR, Windham MD (1991) A model for divergent, allopatric speciation of polyploid Pteridophytes resulting from silencing of duplicate-gene expression. *Am Nat* 137(4):515–526.

[14] Lynch M, Force A (2000) The probability of duplicate gene preservation by subfunctionalization. *Genetics* 154(1):459–473. 10629003 10.1093/genetics/154.1.459 PMC1460895

[15] Muller HJ (1942) Isolating mechanisms, evolution and temperature. *Biol Symp* 6:71–125

[16] Dobzhansky T (1937) *Genetics and the Origin of Species*. New York: Columbia University Press.

[17] Masly JP, Presgraves DC (2007) High-resolution genome-wide dissection of the two rules of speciation in *Drosophila*. *PLoS Biol* 5(9):e243 doi: 10.1371/journal.pbio.0050243 17850182 10.1371/journal.pbio.0050243 PMC1971125

[18] Bikard D, Patel D, Le Metté C, Giorgi V, Camilleri C, Bennett MJ et al (2009) Divergent evolution of duplicate genes leads to genetic incompatibilities within *A*. *thaliana*. *Science* *(80-)* 323(5914):623–626. doi: 10.1126/science.1165917 19179528 10.1126/science.1165917

[19] Bank C, Bürger R, Hermisson J (2012) The limits to parapatric speciation: Dobzhansky-Muller incompatibilities in a continent-Island model. *Genetics* 191(3):845–863. doi: 10.1534/genetics.111.137513 22542972 10.1534/genetics.111.137513 PMC3389979

[20] Höllinger I, Hermisson J (2017) Bounds to parapatric speciation: A Dobzhansky–Muller incompatibility model involving autosomes, *X* chromosomes, and mitochondria. *Evolution (N Y)* 71(5):1366–1380. 10.1111/evo.13223 28272742

[21] Turissini DA, Matute DR (2017) Fine scale mapping of genomic introgressions within the *Drosophila yakuba* clade. *PLoS Genet* 13(9):e1006971 doi: 10.1371/journal.pgen.1006971 28873409 10.1371/journal.pgen.1006971 PMC5600410

[22] Ferris KG, Barnett LL, Blackman BK, Willis JH (2017) The genetic architecture of local adaptation and reproductive isolation in sympatry within the *Mimulus guttatus* species complex. *Mol*. *Ecol*, 26: 208–224. doi: 10.1111/mec.13763 27439150 10.1111/mec.13763

[23] Wang RJ, Hahn MW (2018) Speciation genes are more likely to have discordant gene trees. bioRxiv. doi: 10.1101/244822 10.1002/evl3.77 PMC6121824 30283682

